# Error of Position

**Published:** 1855-05

**Authors:** 


### BIBLIOGRAPHICAL NOTICES.

### ERROR OF POSITION.

This is a pamphlet published at Nashville, Tennessee, by some one who styles himself Prof. Milo, which we presume is equivalent to an anonymous signature. Its pages are ornamented with an obscene cut, representing an individual looking backward with his head down between the thighs,—usually a very unfortunate position, but doubtless a natural one, if the cut be a delineation of the author himself. *Other* structures, than those believed to be the instrument of the mind, are here represented as most prominent, between which, and the production before us, there is certainly a natural relation. We would expect such an emanation from such a *source*. Lot's wife was said to be looking back when she was transformed into that which should remain as a monument of her disobedience, and like it, this cut will not only perpetuate his refined and classical taste, but his vanity and folly.

If that pamphlet was written by a member of the profession, it insults and disgraces it, and the author should be allowed hereafter an oblivious obscurity. We would enquire of our brethren of Nashville, whether there is a Lunatic Asylum in the State, in which, if the author were to find a place, there would be no "*error of position*?"

